# Progress in Surgery

**Published:** 1896-12-19

**Authors:** 


### PROGRESS IN SURGERY.

### SURGERY OF THE ŒSOPHAGUS AND STOMACH.

**Foreign Bodies in the Œsophagus** are difficult to detect when impacted, but skiagraphy has recently been shown to be of service in a case of Taubet.[1] A child, aged two years and five months, began to complain of pain in swallowing, and rejected or vomited all her food. It was found that one of a set of "jack-stones" was missing, and on exposing the thorax to the Röntgen rays for three minutes the plate showed the missing stone to be in the œsophagus, on a level with the space between the second and third dorsal spines. It was not possible to remove it with forceps, so gastrotomy was performed, and by means of pledgets of gauze attached to a piece of string passed through the mouth, down the œsophagus and out at the gastrotomy wound, the body was dislodged into the stomach, and was then easily extracted. The child made a good recovery.

**Gastropexy.**—Duret[2] having found certain dyspeptic symptoms associated with prolapse (described by Glenard under the name of gastroptosis) of the stomach, proposes fixation of the organ, and has in one case—a woman aged fifty-one years—successfully carried out this idea. A median incision, about four inches long, is made in the anterior abdominal wall in the epigastric region. The lower half only of the exposed parietal peritoneum is incised in this wound, the upper portion being left intact for the insertion of the sutures, by which it is proposed to elevate and fix the displaced stomach. By carrying the sutures alternately through the coats of the stomach and the parietal peritoneum three or four times, a large surface of the anterior gastric wall may be brought into wide and close contact with the anterior abdominal wall. Gastropexy must be a rare operation, as Glenard's disease is not of frequent occurrence. In a case complicated with marked displacement of the mass of intestine, it would be easy to associate with the operation on the stomach an enteropexy of the transverse colon or small intestines by stitching up a fold of the elongated mesentery.

**Gastrostomy.**—Marwedel[3] recommends a modification of gastrostomy, resembling Fischer's intra-mural canal. An oblique incision is made on the left side below the ribs, the parietal peritoneum is attached to the skin with catgut, and a portion of the anterior stomach wall drawn out and sutured into the wound. An opening is now made into the stomach, immediately, or after the lapse of a few days, about two or three inches long, going through the serous and muscular layers without injuring the mucosa. A flap of sero-muscular tissue is now dissected up on each side of the incision for about a quarter or one-third of an inch. A small transverse incision is then made through the mucosa near the lower angle of the wound, and through it a thin drainage tube or Jacque's catheter is introduced and fixed into the mucosa by catgut. The serous and muscular flaps are united over the drainage tube. The patient is fed through the tube for four days, after which the tube is removed and introduced for each feeding. An oblique canal forms in the stomach wall between the mucous membrane on one side and the sero-muscular layer on the other, through which large-sized metal instruments can be introduced. The external wound heals up except the fistula, no apparatus being required, and there is no leakage of stomach contents. Speaking of different methods of gastrostomy, Chavasse[4] says that Hahn's method (in which a portion of stomach is brought out through the eighth left intercostal space) appears to give rise to more pain than the ordinary operation, and that it is possible to wound the pleura or the diaphragm during the operation, and that necrosis of the cartilages of the ribs may subsequently supervene. He quotes Kocher[5] and Willie Meyer[6] as giving their preference to Albert's operation—in which a portion of the stomach is drawn out under a bridge of skin. Comparing the plans advocated by Witzel and Albert respectively, the author thinks that one operation is as readily performed as the other, but that in the latter (Albert's) cases occur in which some difficulty will be encountered in drawing the stomach forwards and upwards, owing to the cardiac end of the organ becoming more or less fixed by the neoplasm. The traction on the stomach wall in Albert's operation causes more gastric and general disturbance than Witzel's method. The opening into the viscus is also apt to contract somewhat under the bridge of skin. When complete, however, it has an advantage over Witzel's method in that no tube need be worn. Both methods give satisfactory results as regards leakage, thereby saving much physical discomfort to the patient, which followed the older methods of operation. Bond[7] describes a method which he has found useful in permanently closing the opening after gastrostomy and enterostomy. An oval disc of sheet india-rubber, larger than the fistula, is coiled up and inserted through the fistulous opening by means of a pair of forceps; before passing it into the stomach it is pierced by a double row of plated wire sutures, six or eight in number, each suture going completely through the disc twice and forming a loop on the back. The free ends are loosely held while the plate is let into the stomach, and after unfolding it is drawn up flat against the mucous membrane by the double row of sutures; the edges of the fistula are freshened, care being taken to cut away the everted mucous membrane. By means of a handled needle, each wire is threaded separately and brought through the coats of the stomach and the abdominal wall from within outwards, close to the edge of the opening. In this way one row of sutures passes through one side of the opening, and the other row through the other; the edges can then be brought together and the sutures tied externally. The opening is thus closed against leakage by the button within the cavity. Each suture can be cut and drawn out whole, and the plate allowed to drop into the stomach or the intestinal canal.

**Gastrorrhaphy** for dilated stomach has been proposed and performed with some degree of success in one case by Ewart and Bennett.[8] The indications for this operation are somewhat the same as those for which gastropexy (*vide supra*) has been suggested. After the usual parietal incision, the stomach was drawn out and laid upon the abdomen. A fold on the anterior aspect of the viscus was then turned in by the fingers of the assistant, the length of the fold being about twelve inches, and its depth about three inches at its widest part. The peritoneal surfaces on the opposite sides of this fold were then fastened together by means of sutures transfixing the peritoneal and muscular coats. By this means the size of the stomach was greatly lessened, and the altered viscus was then returned into the abdomen. In extreme cases of gastrectasis this operation is not out of proportion to the benefit likely to ensue.

**Carcinoma of the Stomach.**—Krönlein[9] reports sixty-seven cases. An operation was declined in seven cases, and could not be recommended in nineteen; the average survival of the former class was 209 days, and of the latter 77 days. Of twenty-two cases treated by exploratory incision only, nineteen survived on the average 139 days, and there was a remarkable subjective improvement in their condition. Gastro-enterostomy he limits to such instances as show unmistakable signs of stenosis, for the average mortality being about 43 per cent. is too high, even if palliation was invariably afforded. Resection of the pylorus was performed in fifteen cases only. Eleven cases recovered, four died. Of the survivors, two died of other diseases, four of recurrence—living on the average 597 days—and five are alive, without evident recurrence. The author warns against excessive preparatory use of purgatives or washing out of the stomach before operation. Gibson and Thomson[10] report a successful case of pylorectomy for carcinoma in which, after excision of the tumour, the posterior walls of the stomach and duodenum were united by means of Murphy's button. Lyman[11] has removed a pedunculated carcinoma of the stomach by gastrotomy in a patient aged sixty years. The operation was undertaken with the idea of performing pylorectomy or gastro-enterostomy. Owing to the extent of the disease, any resection of the stomach wall was found to be impossible, and as the interior of the stomach was almost completely filled by a large mass of growth projecting into it, the only means of relieving the patient was by the operation referred to. The patient, however, only lived for about four weeks after operation. A successful case of gastro-jejunostomy with the Murphy button is reported by Cordier,[12] but the diagnosis of malignant disease seems doubtful.

[1] Annals of Surgery, Aug., 1896, p. 238. [2] Brit. Med. Journ., July 25th, 1896, and Revue de Chirurg., June, 1896. [3] Amer. Med. Surg. Bullet., Sept. 5th, 1896, p. 277, and Münch. Med. Woch., 1896, No. 28, p. 665. [4] Lancet, July 4th, 1896, p. 5. [5] Text-book of Operative Surgery, second edition, translated by Stiles. [6] Annals of Surgery, Vol. xviii., p. 558. [7] Brit. Med. Journ, July 4th, 1896. [8] Lancet, July 4th, 1896, p. 8. [9] Brit. Med. Journ., March 21st, 1896; Brun's Beitr. Bd. xv., p. 315; and Edin. Med. Journ., Oct., 1896, [10] Edin. Med. Journ., Oct., 1896. [11] Annals of Surg., Sept., 1896. [12] Med. Rec. New York, Sept. 5th, 1896.

